# Virulence-associated genes and antibiotic susceptibility among vaginal and rectal *Escherichia coli* isolates from healthy pregnant women in Poland

**DOI:** 10.1007/s12223-018-0598-z

**Published:** 2018-04-12

**Authors:** Agnieszka Kaczmarek, Krzysztof Skowron, Anna Budzyńska, Eugenia Gospodarek-Komkowska

**Affiliations:** Department of Microbiology, Ludwik Rydygier Collegium Medicum in Bydgoszcz, Nicolaus Copernicus University in Torun, 9 M. Skłodowskiej-Curie Street, 85-094 Bydgoszcz, Poland

## Abstract

Vaginal and/or rectal *Escherichia coli* colonization of pregnant women is sometimes associated with neonatal infections. Despite the relevance of these strains, they have been rarely described before. Thus, the aim of this study was to compare vaginal (VEC) and rectal *E. coli* (REC) isolates in respect of antimicrobial susceptibility and the frequency of virulence-associated genes (VAGs). The antimicrobial susceptibility of 50 VEC and 50 REC isolates was performed by using the disc diffusion method, and VAGs were detected by PCR. There were no significant differences in the antimicrobial resistance between VEC and REC. Both VEC and REC isolates were mostly resistant to ticarcillin (36 and 30%) and ampicillin (36 and 22%). None of the tested isolates was positive for ESBL. Gene’s *fimH*, *fimA*, *sfa/foc*, *iutA*, *ibeA*, *hlyF*, and *neuC* were detected, respectively, in 98, 92, 32, 28, 12, 8, and 2% of VEC and in 94, 72, 12, 34, 8, 10, and 8% of REC isolates. The co-occurrence of *fimA/H* and *sfa/foc* genes was significantly more prevalent among VEC isolates, in comparison to REC isolates. The study indicated that VEC and REC isolates are quite similar in terms of antimicrobial non-susceptibility and VAGs.

## Introduction

*Escherichia coli* is a major component of the normal commensal microbiota in the human intestinal tract. As a commensal, it contributes to its host’s health. However, some *E. coli* strains can cause variety of infectious diseases (Bagger-Skjøth et al. 2007; Fakhreddin et al. 2013; Hilbert et al. 2008). *E. coli* is one of the main causative agents of gastrointestinal and extra intestinal infections (Obata-Yasuoka et al. 2002). Although pathogenic *E. coli* bacteria have been more commonly recognized as intestinal pathogens, extra intestinal *E. coli* infections, mainly neonatal bacterial meningitis (NBM) and sepsis (NBS). Pathogenic *E. coli* bacteria are also a major source of morbidity and mortality (Kolchak et al. 2005). The ability of *E. coli* to cause these devastating diseases in the neonate depends largely on several virulence factors (VFs), which help to survive under adverse conditions (Kolchak et al. 2005; Obata-Yasuoka et al. 2002; Soto et al. 2008; Watt et al. 2003). Capacity of *E. coli* to produce VFs, including, e.g., K1 capsular antigen, adhesions (P, type 1, F1C, and S fimbriae), IbeA, Mop, Salad and Taj proteins, O-lipopolysaccharide (O-LPS), cytotoxic necrotizing factor 1 (CNF1), the iron uptake aerobatic system, and hemolysis, may contribute to its pathogenicity in NBM and NBS (Binge et al. 1997; Chouikha et al. 2008; Johnson et al. 2002; Kim 2003; Korhonen et al. 1985; Watt et al. 2003). These diseases can lead to death and among more than half of the survivors to serious neurological complications such as seizure disorders, hydrocephalus, physical disability, developmental delay, and hearing loss (Korczak et al. 2005). Neonatal infections often result from maternal transmission of *E. coli* during birth (Korczak et al. 2005; Soto et al. 2008; Watt et al. 2003). Therefore, colonization of the mother’s vagina or rectum by extraintestinal pathogenic *E. coli* (ExPEC), which possesses specialized VFs, uncommon among commensal *E. coli*, plays potentially important role in the development of NBM and NBS (Hilbert et al. 2008; Watt et al. 2003). Besides, the knowledge about antimicrobial susceptibility of vaginal (VEC) and rectal (REC) *E. coli* strains obtained from pregnant women is necessary to select the correct antibiotic(s) for proper treatment of infections caused by them (Fakruddin et al. 2013; Hilbert et al. 2009).

The objective of the present study was to compare VEC and REC isolates in respect of the antimicrobial susceptibility and the frequency of selected virulence-associated genes encoding VFs which are required for successful penetration into the central nervous system (K1 antigen, type 1 and S fimbriae, IbeA protein) and essential to the survival of invasive isolates of *E. coli* when iron is limiting in the bloodstream (aerobactin and hemolysin).

## Material and methods

### Bacterial isolates

In this study, 50 VEC and 50 REC isolates from 100 healthy pregnant women (one isolate per person) were analyzed. The *E. coli* isolates used in our study were obtained from the collection of the Department of Microbiology of the Ludwik Rydygier Collegium Medicum in Bydgoszcz, Nicolaus Copernicus University in Torun. The used *E. coli* were collected from June to September 2008 at Dr. J. Biziel University Hospital No. 2 of the L. Rydygier Collegium Medicum in Bydgoszcz from healthy pregnant women for the prevention of perinatal diseases. The isolates were identified based on colony morphology and cultural characteristics on MacConkey agar and by standard biochemical tests (ID 32E, bioMérieux).

### DNA extraction

DNA was extracted from bacteria using a Genomic Mini Purification kit (A&A Biotechnology) according to the manufacturer’s instructions. Extracted DNA was stored at − 20 °C.

### Detection of virulence-associated genes

Virulence-associated genes (*fimA*, *fimH*, *sfa/foc*, *neuC*, *hlyF*, *iutA*, and *ibeA*) were examined by the multiplex PCR as described previously (Kaczmarek et al. 2012).

### Antimicrobial susceptibility testing

The antimicrobial susceptibility testing of the isolates was performed using the Kirby–Bauer disc diffusion method (Bauer et al. 1966). The antibiotics used in our research were selected in accordance with recommendations of the European Committee on Antimicrobial Susceptibility Testing (2014). The following antimicrobial agent disks were used: ampicillin (10 μg), ampicillin-sulbactam (10–10 μg), amoxicillin-clavulanic acid (20–10 μg), piperacillin (30 μg), piperacillin-tazobactam (30–6 μg), ticarcillin (75 μg), ticarcillin-clavulanic acid (75–10 μg), cefepime (30 μg), cefotaxime (5 μg), cefoxitin (30 μg), ceftazidime (10 μg), ceftriaxone (30 μg), cefuroxime (30 μg), doripenem (10 μg), ertapenem (10 μg), imipenem (10 μg), meropenem (10 μg), aztreonam (30 μg), ciprofloxacin (5 μg), levofloxacin (5 μg), moxifloxacin (5 μg), norfloxacin (10 μg), ofloxacin (5 μg), amikacin (30 μg), gentamicin (10 μg), netilmicin (10 μg), tobramycin (10 μg), tigecycline (15 μg), chloramphenicol (30 μg), and trimethoprim-sulfamethoxazole (1.25–23.75 μg). Standard inocula of tested isolates adjusted to 0.5 McFarland were swabbed on Mueller Hinton agar (Becton Dickinson). Inoculated plates with antibiotic disks were incubated at 35 °C for 18–24 h under aerobic conditions. Then the inhibition zones were measured and interpreted according to the EUCAST. Standard strain of *E. coli* ATCC 25922 was used as a control.

Extended-spectrum beta-lactamases (ESBL) production by *E. coli* isolates was determined by double-disc synergy test (DDST) (Drieux et al. 2008; Jarlier et al., 1988). DDST was performed with cefotaxime (30 μg) and ceftazidime (30 μg) disks placed at a distance of 20 mm (center to center) from the amoxicillin-clavulanic acid disk (20/10 μg). Moreover, cefpodoxime (10 μg) and aztreonam (30 μg) disks were added to increase the sensitivity of the DDST. Additionally, in the same culture medium, cefepime (30 μg) disk was placed, in order to improve the detection of ESBL when the simultaneous stable hyperproduction of a AmpC beta-lactamase occurs. The test result was considered positive when an enhancement of the inhibition zone around at least one of the antibiotic disks (cefotaxime, ceftazidime, cefpodoxime, aztreonam, or cefepime) toward the clavulanic acid disk was observed. Control strains *Klebsiella pneumoniae* ATCC 700603 (ESBL positive) and *E. coli* ATCC 25922 (ESBL negative) were used for quality control.

Multidrug-resistant (MDR) *E. coli* was defined as non-susceptibility to at least one agent in > 3 antimicrobial categories (Magiorakos et al. 2012).

### Statistical analysis

Statistical analysis was conducted by using *χ*^2^ test. The level of significance was set at a *P* < 0.05. Data analyses were performed by Statistica version 10.0 (StatSoft, Poland).

Moreover, the association between antimicrobial resistance and virulence-associated genes was determined. For this purpose, the Pearson correlation coefficient (r) was calculated and its value was referred to the Stanisz scale. Also, the statistical significance of the Pearson correlation coefficient was tested.

## Results

### Frequency of virulence-associated genes

Among the tested isolates, the genes encoding adhesive subunit FimH (96%) and the protein subunit FimA (82%) of type 1 fimbriae occurred the most frequently. Other examined genes, according to the prevalence, can be arranged in descending order: *iutA* (31%), *sfa*/*foc* (22%), *ibeA* (10%), *hlyF* (9%), and *neuC* (5%). Genes *iutA*, *hlyF*, and *neuC* occurred more frequently in REC isolates, while *sfa*/*foc* and *ibeA* in VEC isolates (Table 1). Statistical analysis showed significant differences (*P* < 0.05) in the prevalence of genes *fimA* and *sfa*/*foc* among the examined isolates of *E. coli* due to the place of their isolation (rectum/vagina).

**Table 1:** Prevalence of virulence-associated genes among VEC and REC isolates

Gene	Total (%)	No. VEC (%)	No. REC (%)	*P* value*
*fimA*	82 (82)	*46 (92)*	*36 (72)*	*0.009*
*fimH*	96 (96)	49 (98)	47 (94)	0.307
*iutA*	31 (31)	14 (28)	17 (34)	0.517
*sfa/foc*	22 (22)	*16 (32)*	*6 (12)*	*0.001*
*ibeA*	10 (10%)	6 (12)	4 (8)	0.505
*hlyF*	9 (9%)	4 (8)	5 (10)	0.727
*neuC*	5 (5%)	1 (2)	4 (8)	0.169

*Statistically significant values are marked as italic

### Profiles of virulence-associated genes in VEC and REC isolates

The majority of the analyzed VEC and REC isolates contained two determinants: the *fimA* and *fimH*. Such genetic profile was observed in 31 (31%) isolates, of which 15 (48%) were REC isolates, and 16 (52%) were VEC isolates. The absence of the genes encoding subunits of type 1 fimbriae was demonstrated only for 2 (2%) isolates. Besides *fimA* and *fimH*, the co-occurrence of *fimA*, *fimH*, and *iutA* (16%) and *fimA*, *fimH*, and *sfa*/*foc* (14%) was the most frequently reported. The last of the mentioned profiles occurred statistically significantly (*P* < 0.05) more frequently in VEC than in REC isolates.

Mostly the examined isolates of *E. coli* contained 3 (40%) or 2 (34%) genes encoding selected VFs. Among 3 (3%) isolates, the co-occurrence of 5 genes was found, whereas among 12 (12%) *E. coli* isolates only one gene was detected. None of the tested isolates had all 7 genes. For one isolate, there was no virulence-associated gene detected.

### Analysis of the antimicrobial susceptibility

All the analyzed VEC and REC isolates were susceptible to amoxicillin-clavulanic acid, piperacillin-tazobactam, cefepime, ertapenem, imipenem, meropenem, aztreonam, amikacin, netilmicin, and tigecycline. VEC and REC isolates were intermediate to piperacillin, ciprofloxacin, levofloxacin, moxifloxacin, norfloxacin, ofloxacin, piperacillin, ceftriaxone, doripenem, and tobramycin, respectively (Table 2). Out of all tested isolates, most were resistant to ticarcillin (33%), ampicillin (29%), and piperacillin (26%). The resistance to other groups of antibiotics than penicillins was also observed. Among cephalosporins, the majority of VEC and REC isolates were resistant to cefuroxime (4%), among aminoglycosides—to gentamicin (3%), among fluoroquinolones—to ofloxacin (2%), and among miscellaneous agents—to trimethoprim-sulfamethoxazole (10%) (Table 2). No significant difference in the antimicrobial resistance between VEC and REC isolates was observed (*P* > 0.05).

**Table 2:** Comparison of the antimicrobial non-susceptibility between VEC and REC isolates

Antibiotics	Total (%)		No. VEC (%)		No. REC (%)	
	*R*	*I*	*R*	*I*	*R*	*I*
AM	29 (29)	0 (0)	18 (36)	0 (0)	11 (22)	0 (0)
SAM	9 (9)	0 (0)	7 (14)	0 (0)	2 (4)	0 (0)
PIP	26 (26)	4 (4)	17 (34)	1 (2)	9 (18)	3 (6)
TIC	33 (33)	0 (0)	18 (36)	0 (0)	15 (30)	0 (0)
TIM	17 (17)	0 (0)	12 (24)	0 (0)	5 (10)	0 (0)
FOX	1 (1)	0 (0)	0 (0)	0 (0)	1 (2)	0 (0)
CTX	2 (2)	0 (0)	0 (0)	0 (0)	2 (4)	0 (0)
CAZ	3 (3)	0 (0)	1 (2)	0 (0)	2 (4)	0 (0)
CRO	2 (2)	1 (1)	0 (0)	0 (0)	2 (4)	1 (2)
CXM	4 (4)	0 (0)	1 (2)	0 (0)	3 (6)	0 (0)
DOR	0 (0)	1 (1)	0 (0)	0 (0)	0 (0)	1 (2)
CIP	0 (0)	1 (1)	0 (0)	1 (2)	0 (0)	0 (0)
LVL	0 (0)	3 (3)	0 (0)	3 (6)	0 (0)	0 (0)
MXF	1 (1)	1 (1)	1 (2)	1 (2)	0 (0)	0 (0)
NOR	0 (0)	3 (3)	0 (0)	3 (6)	0 (0)	0 (0)
OFX	2 (2)	3 (3)	2 (4)	3 (6)	0 (0)	0 (0)
GM	3 (3)	0 (0)	0 (0)	0 (0)	3 (6)	0 (0)
NN	0 (0)	3 (3)	0 (0)	2 (4)	0 (0)	1 (2)
C	5 (5)	0 (0)	2 (4)	0 (0)	3 (6)	0 (0)
SXT	10 (10)	0 (0)	3 (6)	0 (0)	7 (14)	0 (0)

*R* resistant, *I* intermediate, *AM* ampicillin, *SAM* ampicillin-sulbactam, *PIP* piperacillin, *TIC* ticarcillin, *TIM* ticarcillin-clavulanic acid, *FOX* cefoxitin, *CTX* cefotaxime, *CAZ* ceftazidime, *CRO* ceftriaxone, *CXM* cefuroxime, *DOR* doripenem, *CIP* ciprofloxacin, *LVL* levofloxacin, *MXF* moxifloxacin, *NOR* norfloxacin, *OFX* ofloxacin, *GM* gentamicin, *NN* tobramycin, *C* chloramphenicol, *SXT* trimethoprim-sulfamethoxazole

None of the tested isolates was positive for ESBL.

### Resistance profiles in VEC and REC isolates

More than half (59%) isolates analyzed in this work were susceptible to all tested drugs. The results showed that the remaining 41% of VEC and REC isolates were resistant or intermediate to at least one antimicrobial agent. There was no significant association between antimicrobial resistance patterns and site of isolation (*P* > 0.05).

Resistance to 1–3, 4–5, and 6–8 antibiotics was reported in 10 (27.1%), 22 (59.4%), and 5 (13.5%) isolates, respectively. Isolates resistant to 7–8 antibiotics were found only among REC. Three (6%) and 5 (10%) isolates were classed as MDR strains among VEC and REC, respectively.

### Association between antimicrobial resistance and virulence-associated genes

Both for VEC and REC isolates, the conducted analysis has revealed statistically insignificant (*P* > 0.05) but slightly positive correlation between the number of antibiotics to which the tested isolates were resistant and the number of the genes encoding VFs, which they possessed. For the VEC isolates, the Pearson correlation coefficient was 0.23 (*P* = 0.11) and for REC isolates was 0.13 (*P* = 0.39). It follows that there was weak tendency toward increasing the virulence of isolates with the increase of their antibiotic resistance.

## Discussion

The digestive tract is a natural reservoir of *E. coli*. These strains are probably the source of colonization of the vagina. The presence of *E. coli* strains on mucous membranes of the genital tract and/or anus of pregnant women may lead to infection of a newborn during labor, especially to the development of meningitis, as well as bacteremia, sepsis, and urinary tract infections (Bingen et al. 1997; Korczak et al. 2005; Obata-Yasuoka et al. 2002; Watt et al. 2003).

Specific interactions between bacterial fimbriae containing adhesins and eukaryotic cell receptors play a key role in adhesion of *E. coli* strains and other Gram-negative bacteria to the host cell surface. *E. coli* produce many types of fimbriae, of which type 1 fimbriae often occur on their surface. The type 1 fimbriae are encoded by a *fim* gene cluster, including nine genes required for its biosynthesis (Pusz et al. 2014). We screened for the presence of *fimA* gene encoding the major subunit of type 1 fimbriae and the presence of *fimH* gene that determines the biosynthesis of the specific adhesin of these fimbriae. The occurrence of *fimA* gene was found in 92% of VEC isolates and in 72% REC isolates. Whereas, *fimH* gene was found in 98 and 94% of the studied VEC and REC isolates, respectively. Identification of *fimA* in a lower percentage than *fimH* (especially among REC isolates) confirmed the results obtained by Pusz et al. (2014). Authors showed that many of *E. coli* strains were carrying an incomplete set of genes in the *fim* gene cluster. Moreover, their research indicated that among commensal *E. coli* strains the absence/deletion of *fimA* gene is most commonly observed. Possibly, incomplete set of genes responsible for the type 1 fimbriae expression is an adaptive feature of *E. coli* to the habitation of the human intestine environment. Other authors indicated the presence of *fimA* and *fimH* among 60–100% of VEC and REC strains (Hilbert et al. 2008; Moreno et al. 2008; Obata-Yasuoka et al. 2002; Pusz et al. 2014; Watt et al. 2003).

*E. coli* also possess S fimbriae, which apart from adhesin FimH, are a substantial factor enabling *E. coli* adhesion to brain microvascular endothelial cells (BMEC) and overcoming the blood-brain barrier (Kim 2003). The presence of these fimbriae is most often found in *E. coli* strains isolated from bacteremia, NBS, and NBM cases. Johnson et al. (2002) proved that operon *sfa/foc*, that encodes the synthesis of S fimbriae and F1C fimbriae, occurs in 61% *E. coli* isolated from neonates with meningitis. S and F1C fimbriae are also a common virulence factor among VEC strains. The authors (Hilbert et al. 2008; Obata-Yasuoka et al. 2002; Watt et al. 2003) report that these fimbriae occur in 20–48% of VEC strains. Their occurrence is less often (2–28%) noted among *E. coli* derived from feces (Bagger-Skjøt et al. 2007; Bingen et al. 1997; Cook et al. 2001; Duriez et al. 2001; Hilbert et al. 2008; Korhonen et al. 1985; Moreno et al. 2008; Obata-Yasuoka et al. 2002; Watt et al. 2003). Similarly, in the present study the percentage of isolates having the *sfa/foc* gene was significantly higher among VEC isolates (32%) as compared with the group of REC isolates (12.0%).

Adhesion and subsequent penetration of pathogenic *E. coli* strains into the cells forming the blood-brain barrier is necessary for these bacteria to induce meningitis. This process involves, among others, participation of membrane proteins IbeA (Che et al., 2011; Kim 2003). In the present study, the *ibeA* gene occurred in a low percentage of both VEC and REC isolates, while it was more often present in VEC isolates (12%) than in REC isolates (8%). Higher frequency of the *ibeA* gene among VEC than REC strains was also shown by Obata-Yasuoka et al. (2002) (32 vs 26%) and Watt et al. (2003) (20 vs 4%).

An essential virulence factor of *E. coli* strains is K1 antigen. Many authors (Bingen et al. 1997; Johnson et al. 2002; Korhonen et al. 1985; Obata-Yasuoka et al. 2002; Watt et al. 2003) have indicated that 78–92% of neonatal meningitis *E. coli* strains (NMEC) have K1 antigen. K1 antigen less often occurs in VEC and REC strains. Watt et al. (2003) found that 44% of VEC strains isolated from the pregnant women have *neuC* gene encoding K1 antigen. A similar percentage of VEC strains with K1 antigen was reported by Obata-Yasuoka et al. (2002) both in pregnant women (40%) and non-pregnant women (48%). In the present study, the presence of the *neuC* gene was found only in 1 out of 50 examined VEC isolates. In turn, colonization of the rectum by *E. coli* K1 strains is found among 45–50% women (Feigin et al. 1992). Watt et al. (2003) proved the presence of *neuC* gene in 42% of REC strains of pregnant women. In contrast, in this work *neuC* gene was only detected in 8% of the examined REC isolates.

The ability to produce hemolysin and aerobactin is also of utmost importance for *E. coli* pathogenicity. In the present study, no statistically significance differences were observed between compared groups of VEC and REC isolates in the frequency of *iutA* (28 vs 34%) and *hlyF* (8 vs 10%) genes, encoding the receptor for aerobactin and hemolysin F, respectively. The percentage of VEC isolates with *iutA* gene showed in this study was similar to the one noted by Obata-Yasuoka et al. (2002) (35%). However, other authors, (Guiral et al. 2011; Watt et al. 2003), showed the presence of genes encoding aerobactin (*iutA*, *iucC*) in 41–56% of strains isolated from the vagina and cervix. The frequency of *iutA* gene among REC isolates was comparable with the results obtained by Moreno et al. (2008) (30%), Obata-Yasuoka et al. (2002) (30%), and Watt et al. (2003) (33%) for *E. coli* strains isolated from feces. In the available literature, information about the frequency of *hlyF* gene (hemolysin F) in strains isolated from pregnant women could not be found. The percentage of *E. coli* strains with *hlyF* gene similar to obtained one in this study was obtained in a previous study (Kaczmarek et al. 2017).

In predicting the course of infection, it is important to evaluate the drug susceptibility of bacteria. In this study, the highest percentage of VEC and REC isolates was resistant to ticarcillin (33%), ampicillin (29%), and piperacillin (26%). Similar percentage (30.2%) of isolates resistant to ticarcillin was isolated from pregnant women and neonates in a previous study (Kaczmarek et al. 2017). The percentage of *E. coli* isolates resistant to ampicillin similar to that obtained in this study was reported by Meyn and Hillier (1997) (27%) and Barcaite et al. (2012) (25.9%). However, other researchers reported higher level of non-susceptibility to ampicillin (39.5–65%) (Hilbert et al. 2009; Sáez-López et al. 2016b; Villar et al. 2013) and lower level of resistance to piperacillin (16.6 and 18.6%, respectively) (Barcaite et al. 2012; Kaczmarek et al. 2011).

In this study, the resistance to other antibiotics (especially ticarcillin-clavulanic acid—17% and trimethoprim-sulfamethoxazole—10%) was also observed. In previous studies, no *E. coli* isolate showed resistance to ticarcillin-clavulanic acid, whereas resistance to trimethoprim-sulfamethoxazole was observed in 8.2% of *E. coli* strains with K1 antigen and non-K1 (Kaczmarek et al. 2011). A similar percentage of *E. coli* strains resistant to trimethoprim-sulfamethoxazole was observed in the study by Hilbert et al. (2009). Other authors reported 61% of VEC strains resistant to trimethoprim-sulfamethoxazole (Sáez-López et al. 2016a), probably because of extensively use of drugs as prophylaxis for opportunistic HIV infections in Manhiça.

All VEC and REC isolates were susceptible to amoxicillin-clavulanic acid, piperacillin-tazobactam, cefepime, ertapenem, imipenem, meropenem, aztreonam, amikacin, netilmicin, and tigecycline. Similar results were also obtained in the authors’ earlier study (Kaczmarek et al. 2011, 2017). Similarly, as in this study, Barcaite et al. (2012) revealed that all of pregnant women’s strains were susceptible to amikacin and imipenem and meropenem.

In this and in previous study (Kaczmarek et al. 2011, 2017), no ESBL-positive isolates were detected among the examined *E. coli*. Sáez-López et al. (2016a) found the presence of ESBLs only in one (0.8%) VEC isolate from a pregnant woman. In turn, Villar et al. (2013) revealed that 5.4% of pregnant women were colonized with *E. coli* ESBL-produced strains. Al-Mayahie (2013) showed multidrug resistance in 56.2% of ESBL-producing VEC strains from pregnant and non-pregnant women.

In summary, VEC and REC colonization is related to obstetric infections and the consequent development of infections in newborns. In the present study, it was indicated that VEC isolates are not enriched in antimicrobial non-susceptibility and VAGs relative to the REC. We detected statistically significant differences between these isolates only in the frequency of occurrence of *fimA* and *sfa/foc* genes. Moreover, we observed that the virulence of VEC and REC isolates slightly increased with the increase of their antibiotic resistance.

## Conclusion

VEC or REC colonization may be a risk factor for complications during pregnancy, especially if these strains are drug resistant and have many VAGs resulting in treatment failure or infections. Therefore, the knowledge about the resistance and virulence associated with VEC and REC strains is important in order to determine the probability and the severity of infection these strains may cause.
